# The Improvement of Flaw Detection by the Configuration of Uniform Eddy Current Probes

**DOI:** 10.3390/s19020397

**Published:** 2019-01-18

**Authors:** Ageng S. Repelianto, Naoya Kasai

**Affiliations:** 1 Department of Risk Management and Environmental Sciences, Graduate School of Environmental and Information Sciences, Yokohama National University, Yokohama 240-8501, Japan; 2 Department of Electrical Engineering, Faculty of Engineering, Lampung University, Bandar Lampung 35141, Indonesia

**Keywords:** uniform eddy current, eddy current testing, alternating current field measurement, weld zone, crack, steel, carbon fiber reinforced plastic

## Abstract

In this review, the principles to detect flaws with uniform eddy currents were presented based on the shape and orientation of the excitation coils and detection coils of the probe. Techniques are applied to detect flaws like cracks, especially on the weld zone surface, of test pieces of non-magnetic and ferromagnetic materials, and have unique features which are immune to the effects of lift-off. In the technique of interest, almost all the probe models developed are the type with tangential rectangular excitation coils. The induction condition and the flaw signal for each probe were discussed based on the shape and orientation of the excitation coils and detection coils of the probe. Finally, the challenge of increasing sensitivity to detect flaws with a uniform eddy current was also presented.

## 1. Introduction

The eddy current technique (ECT) is one of the effective non-destructive methods to detect flaws in piping and conductive plat [1,2,3,4]. Over the years, many researchers have applied ECT to detect and evaluate flaws such as cracks [5,6,7,8] on uneven surfaces such as welding zones [9,10,11].

**Figure 1:**
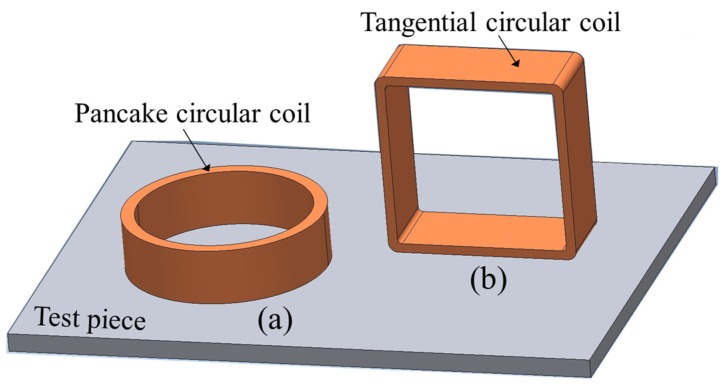
Shape and orientation of coils. (**a**) Pancake circular coil; (**b**) tangential rectangular coil.

Traditional eddy current testing uses a pancake circular-shaped excitation coil, as shown in Figure 1a. It causes a circular eddy current flow pattern. This structure results in strong induction. However, the lift-off effect due to an uneven surface causes a change in impedance, so that it can provide false information regarding the desired inspection parameters [12,13,14,15].

A candidate type of ECT to test pieces with an uneven surface is ECT using a straight-line pattern of an induced eddy current in a detection area, by using a tangential rectangular excitation coil as shown in Figure 1b. In Japan, this technique is known as uniform eddy current (UEC) [16]. Meanwhile, in America and Europe this technique is known as alternating current field measurement (ACFM) [17].

The principle of the methods is similar. The difference is that the UEC excitation core is only the air, while ACFM uses a ferrite core of an excitation coil or magnetizer to obtain a strong magnetic field. The UEC is generally generated by a tangential rectangular excitation coil with a separate probe type.

Compared with pancake coils, tangential coils cause induction to become weak, and the eddy current density produced is also small and only provides a weak detection signal. However, when the lift-off is changed, it experiences a change in amplitude that does not affect the eddy current flow orientation. This condition makes the phase signals more stable. The UEC technique (UECT) is capable detecting flaws on uneven surfaces, such as weld zones. Additionally, it is also able to detect flaws through the coating without removal [3,18,19].

In this paper, we review the principles of self-nulling, and self-differential characteristics and detection of flaws like cracks on the surface of test pieces of non-magnetic and ferromagnetic materials, with several UEC and ACFM probe models presented. In addition, a comparison of the advantages and limitations of each probe is also given. Finally, the challenge of increasing the sensitivity in order to detect flaws with UEC is also presented.

## 2. The Characteristics of Uniform Eddy Current (UEC)

**Figure 2:**
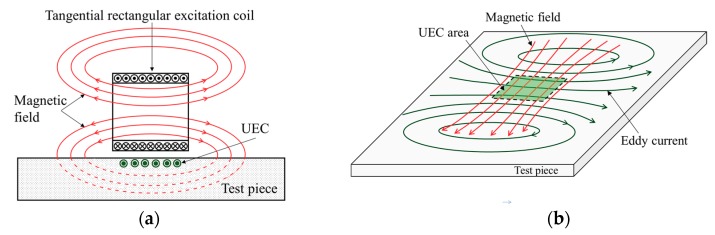

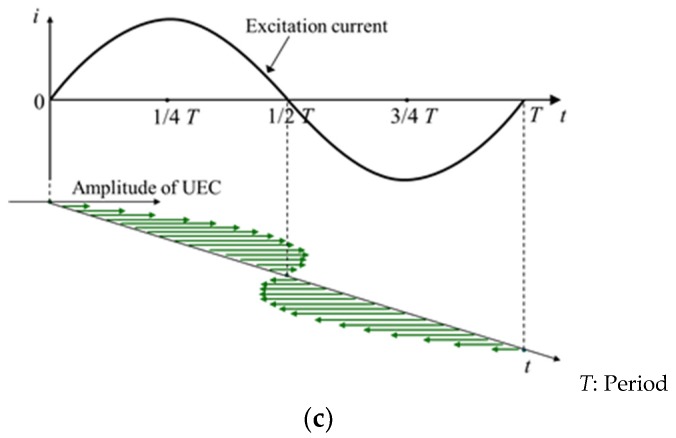
Principle of generation of the uniform eddy current (UEC). (**a**) Sectional view indicating the flow of the UEC and magnetic flux of the tangential excitation coil. (**b**) UEC area on the surface of the test piece. (**c**) Relationship between the excitation current and the amplitude of the uniform eddy current on the UEC area.

Conceptually, the UEC has an eddy current flowing in a straight-line pattern which is generated by the tangential rectangular excitation coil. Figure 2a shows the sectional view, indicating the flow of the UEC and magnetic flux of the tangential excitation coil. A tangential rectangular excitation coil with alternating current supply generates a magnetic field, and the eddy current is induced on the surface of the test piece. The UEC flows on the surface of the test piece, parallel to the winding direction of the excitation coil. The uniform eddy current occurs in straight lines perpendicular to the magnetic fields, which are in a specific area (the UEC area of Figure 2b). As the amplitude of the excitation current changes, the induced eddy currents have a uniform amplitude with one-direction, whose polarity changes every half cycle of the period, as shown in Figure 2c.

Based on the eddy current flow pattern in the test piece, the probe design is divided into two types—one-direction UEC and rotating UEC. The one-direction UEC pattern is produced by a tangential rectangular excitation coil or a magnetizer [20]. Rotating eddy currents can be generated by a combination of two excitation coils or two magnetizers arranged crossed orthogonal to one another. Two excitation currents with a phase difference of 90° are used. Further explanation of both types is in the following section.

## 3. Self-Differential and Self-Nulling Characteristics

A probe using UEC phenomena generally has a unique property whereby the output due to the change of the local conditions of the test piece, such as lift-off, is cancelled. This is called a self-differential characteristic. The property which makes a probe in balance so that the output of a detector coil is zero in normal conditions is known as a self-nulling characteristic.

**Figure 3:**
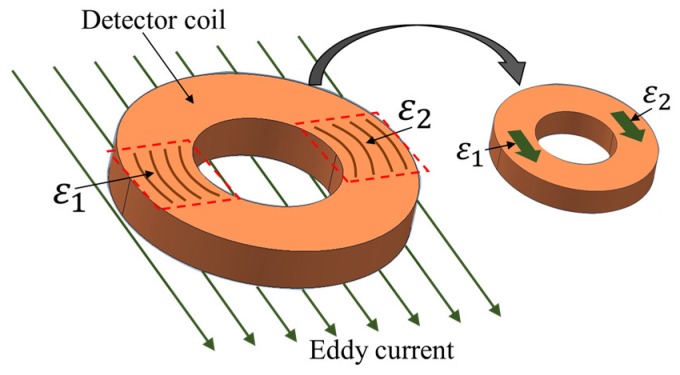
Illustration of self-differential and self-nulling properties.

When the UEC flows under the detector coil, in the part of the coil winding like an arc and parallel to the direction of the eddy current flow arises the electromotive force (EMF) ε  on both parts of the coil, $\varepsilon_{1}$ and $\varepsilon_{2}\;$, as shown by Figure 3. Ideally, the amplitudes of them are the same, but differ in polarity. Hence, they cancel each other out. In balance conditions, the EMF is zero due to the self-nulling property [20]. This is represented in the following equation:

(1) $$\varepsilon =\varepsilon_{1}-\varepsilon_{2}$$

where ε is the EMF of the detector coil, $\varepsilon_{1}$ is the EMF on the left part of the coil, and $\varepsilon_{2}$ is the EMF on the right part of the coil.

Furthermore, when there is lift-off variation, the eddy current will be changing, affecting the EMF on both sides through opposite magnetic fields [21,22]. Even though both the EMF values change, the amplitude of both remains the same. Therefore, the output of the coil remains at zero due to them cancelling each other. The ability to eliminate the influence of lift-off is the critical advantage of the UEC probe technique.

## 4. Apparatus of the UEC System

**Figure 4:**
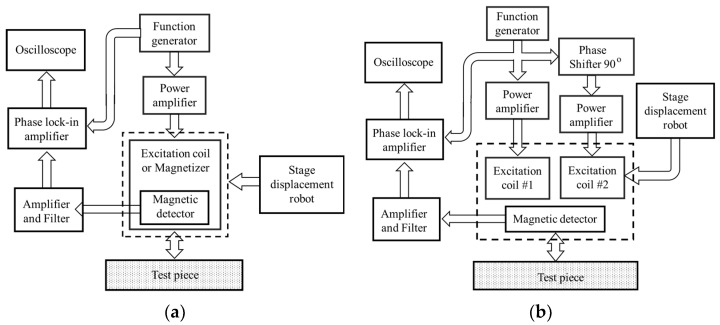
Apparatus of the UEC system. (**a**) One-direction UEC system. (**b**) Rotating UEC system.

There are two types of UEC probe operational support systems, as previously mentioned: apparatus for a one-direction UEC, and apparatus for a rotating UEC. For the one-direction type, the system consists of a function generator, a power amplifier, a UEC probe, a signal amplifier with filter, and a phase lock-in amplifier, as shown in Figure 4a. The function generator is a source of excitation signals. The excitation current with a power amplifier is arranged from an excitation coil and a magnetizer of the UEC probe. Meanwhile, the detection signal generated by the detector coil is quite weak. In order to obtain a measurement signal with a high signal-to-noise (S/N) ratio, the detection signal is amplified and filtered by amplifiers and filter circuits. Finally, the voltage and phase signals are measured to evaluate flaws using a phase lock-in amplifier device. For the sensing process in all surface areas of the test piece, a robot device is required to precisely arrange the displacement position of the probe.

The systems on the rotating UEC probe have the same devices as in the one-direction UEC probe, such as a function generator, a pair of power amplifiers, an amplifier with filter, and a probe with a combination of two excitation coils and a detector coil, arranged as shown Figure 4b. A phase shifter is used to create the 90° phase difference, and two excitation currents supply excitation coil #1 and excitation coil #2, which induce rotating eddy currents [2,16].

## 5. UEC Probe Design Models

### 5.1. One-Direction UEC Probes

#### 5.1.1. One-Direction Hoshi Probe (One Tangential Rectangular Excitation Coil and One Pancake Circular Detector Coil)

**Figure 5:**
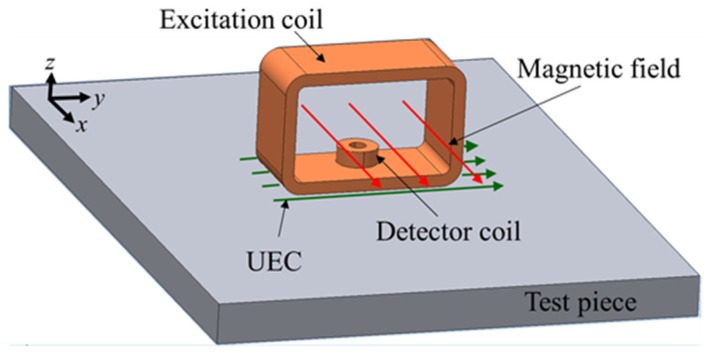
The structure of the One-Direction Hoshi (ODH) probe.

The One-Direction Hoshi (ODH) probe consists of a large tangential rectangular excitation coil and a small pancake circular detector coil whose position is in the lower middle of the excitation, as shown in Figure 5. The ODH probe is designed to be able to detect flaws like cracks in the weld zone, or the rough surface test piece of non-magnetic stainless steel and the edge of the piece. Additionally, it is immune to lift-off noise [10,20]. The unique element proposed from the design of this probe is a detector coil that has self-differential and self-nulling properties, so that it can eliminate the need for a bridge for balancing.

**Figure 6:**
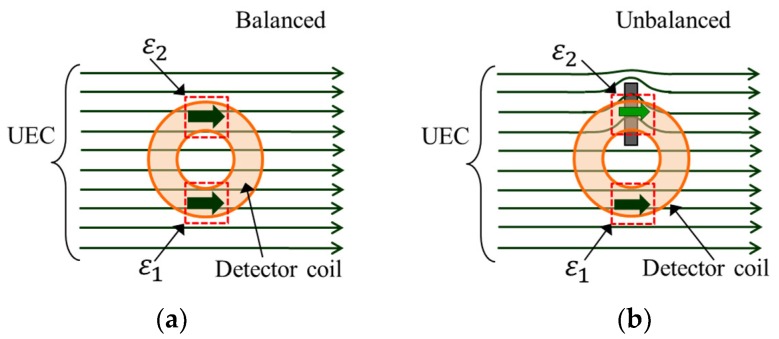
Electromotive force (EMF) conditions are balanced (**a**) and unbalanced (**b**) due to the presence of a flaw.

In the absence of flaws in the test piece, the detector coil with its self-differential and self-nulling properties will keep the ε  at zero, as shown in Figure 6a. When there is a flaw, eddy currents are in disorder due to the flaw and cause the opposite magnetic field to become distorted. This situation is captured by the detector coil as an unbalanced condition, where $\varepsilon_{1}\neq \varepsilon_{2}$. Therefore, ε appears as a representation of the flaw, as shown in Figure 6b.

**Figure 7:**
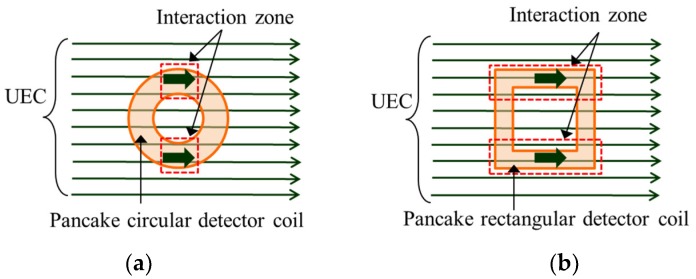
Interaction zone of the detector coils. (**a**) Interaction zone on the pancake circular detector coil. (**b**) Interaction zone on the pancake rectangular detector coil.

One critical factor for increasing sensitivity is to enlarge the interaction zone between the detector coil and the capture area of the opposing magnetic field [20,23,24]. The study was carried out by Hoshikawa on the two forms of detector coils, namely circular and rectangular detector coils, as shown in Figure 7. Both show good performance, withstanding lift-off noise. However, the rectangular detector shows a larger detection signal compared to a circular one, since a rectangular detector coil has larger interaction zone than a circular detector coil.

#### 5.1.2. Cross Probe (One Tangential Rectangular Excitation Coil and One Tangential Rectangular Detector Coil)

**Figure 8:**
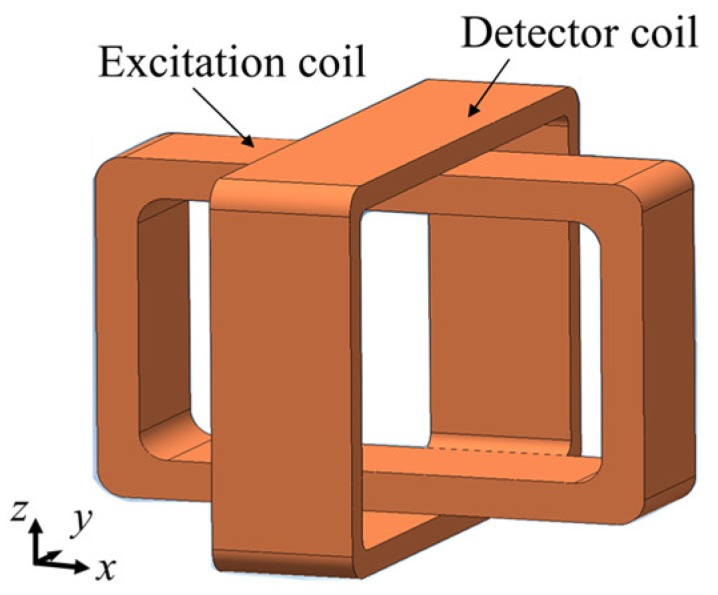
The structure of cross probe.

**Figure 9:**
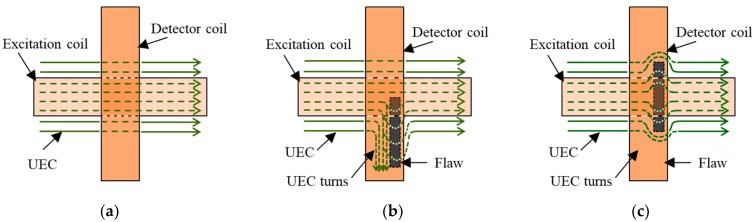
The eddy current flows under a cross probe, (**a**) without a flaw, (**b**) with a flaw, (**c**) with a flaw in the middle of the detector coil.

The other type of the one-direction UEC probe is the cross probe. The probe structure consists of a tangential excitation coil and a tangential detector coil that are upright to each other, as shown in Figure 8. The probe is able to reduce lift-off noise, and since both of the coils are relatively large, the probe has a high S/N ratio signal [25]. The materials of the test pieces are carbon fiber-reinforced composites (CFRP) and steel. The probe can detect flaws like cracks. Without flaws in the test piece, the detector coil does not generate the EMF. Furthermore, if a lift-off occurs, the EMF is kept as a zero value since there is no change of direction of the eddy current flow, as shown in Figure 9a. The probe has self-differential and self-nulling characteristics. The presence of a flaw, as shown in Figure 9b, will distort the eddy current circulation and change the direction of part of the current to parallel with the detector coil winding. As a result, the detector coil generates the EMF. If the flaw position is in the middle of the detector coil, the self-nulling properties cause the EMF on both sides of the detector cancel each other out, so that the EMF is zero, as shown in Figure 9c. This would be a disadvantage for a probe that has self-differential features.

#### 5.1.3. Plus-Probe (One Tangential Rectangular Excitation Coil and Two Tangential Rectangular Detector Coils)

**Figure 10:**
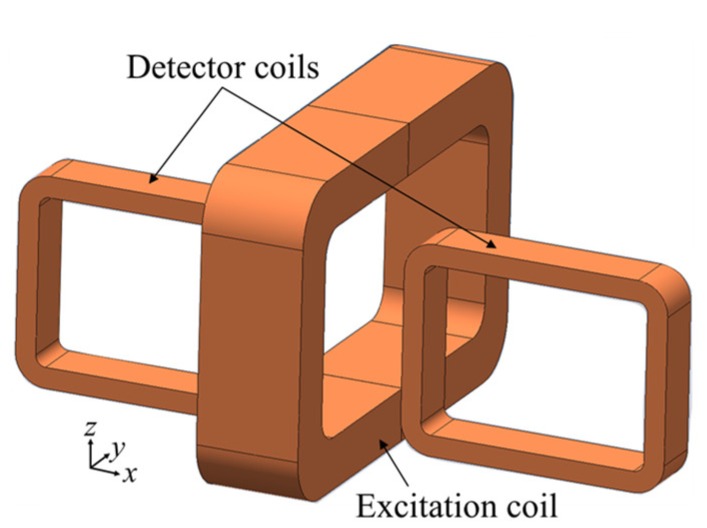
The structure of the Plus probe.

The Plus probe is one type of one-direction UEC probe. The probe consists of an excitation coil and a pair of detector coils, as shown in Figure 10. Both detector coils are orthogonal to the excitation coil, and the large detector coils make high S/N ratio signals due to their large interaction zone. Furthermore, the measured phase signals with the probe are more resistant to lift-off noise [7].

Since the configuration is similar to cross probes, the probe has self-differential and self-nulling properties and EMF occurring due to a flaw being generated by similar phenomena as the cross probe. The detector coils become two separate parts, resulting in more stability in the phase signal.

When scanning a flaw like crack on the surface of the test piece of brass and steel, a flaw signal has two peaks, whose distances show the flaw length.

An increased depth of flaw on the surface of the test piece significantly changes the phase signal. Meanwhile, a change of flaw length with the same depth does not affect the phase of the signal. Thus, flaws area quantitatively evaluated with the Plus probe by using the amplitude and phase of the signal [7].

#### 5.1.4. UEC Probe with a Giant-Magnetoresistance (GMR) Detector (One Tangential Rectangular Excitation Coil and One GMR Detector)

**Figure 11:**
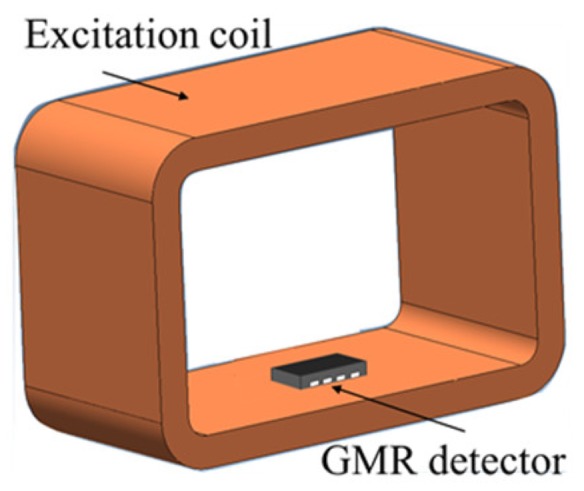
The structure of a tangential rectangle UEC probe with a giant-magnetoresistance (GMR) detector.

The probe structure is quite simple, consisting of a large tangential rectangular excitation coil and a giant-magnetoresistance (GMR) detector, as shown in Figure 11. The GMR detector is installed so that it detects flaws whose lengths are parallel to the flow of the eddy current. Furthermore, the GMR detector is used with low excitation frequencies below 1 kHz, and this capability is one of the advantages of the GMR probe compared to other surface probe models that recommend excitation frequencies above 10 kHz [6,9]. Therefore, it allows detection of subsurface flaws [26,27]. In addition, a GMR with high sensitivity makes it effective for detecting flaws like cracks on aluminum test piece [28].

#### 5.1.5. U-Shaped Alternating Current Field Measurement (ACFM) Probe (One Magnetizer and One Combination of a Tangential Rectangular Coil and a Pancake Rectangular Coil of Detector Coil)

The ACFM was developed by a researcher of University College London for detecting cracks in underwater environments [4].

**Figure 12:**
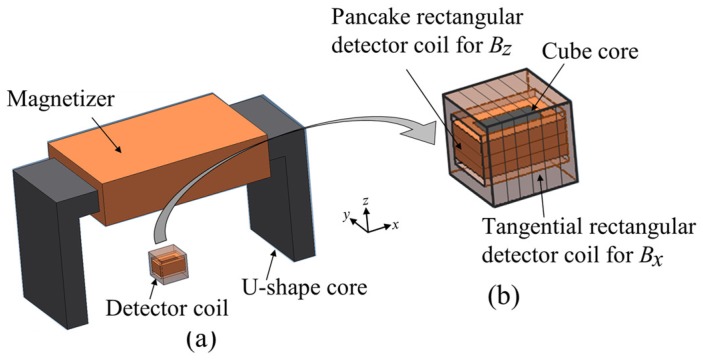
The structure of the one-direction alternating current field measurement (ACFM) probe, (**a**) One-direction ACFM probe. (**b**) Detector coil is a combination of tangential rectangular detector coil for $B_{x}$ and pancake rectangular detector coil for $B_{z}$.

The configuration of the ACFM probe is shown in Figure 12. It has a magnetizer and a combination of a tangential rectangular detector coil to measure the magnetic flux density in the *x* direction ($B_{x}$), and a pancake rectangular detector coil to measure the magnetic flux density in the *z* direction ($B_{z}$) with a cubic ferrite core. The flow of the induced UEC due to the probe is perpendicular to the flow of the magnetic field on the surface of the test piece.

The detector coils simultaneously measure $B_{x}$ and $B_{z}$ at the same position. When there are no flaws in the surface of the test piece, the signals measured with the coils have a constant value. However, if there is a flaw, the $B_{x}$ signal shows two peaks due to a high intensity of UEC at the edges of the flaw. Meanwhile, the $B_{z}$ signal indicates a sunken area that demonstrates the depth of the flaw [22].

The probe can detect flaws like cracks even from through the protective paint and the coating on the surface of the test piece of steel. Since the operators are not required to carry out surface cleaning, it reduces the inspection time [3,17]. The ACFM probe can also be applied to a test piece with a high temperature [2].

#### 5.1.6. IOnic Probe (One Tangential Rectangular Excitation Coil and Two Pancake Semicircular Planar Detector Coils)

The IOnic probe is a new in the design of UEC probes, and has advantages such as not creating heat, low leakage inductance, flexible substrate material [29], and being cheap in terms of production compared to other conventional applications [30]. It also presents increased immunity to the lift-off effect, and enhanced sensitivity [31].

**Figure 13:**
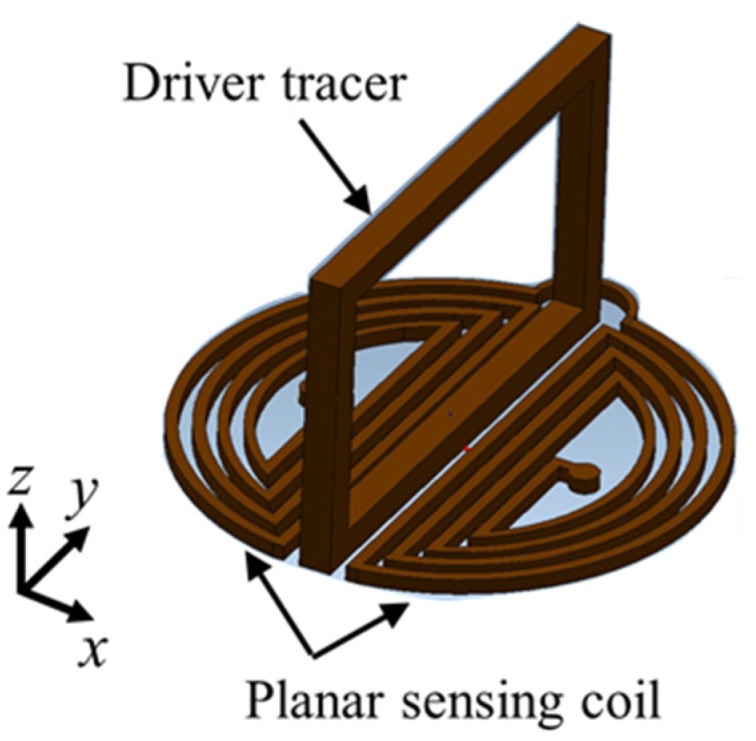
Structure of the IOnic probe.

The IOnic probe consists of an excitation coil, which is termed the driver trace, in the form of a tangential rectangle excitation coil and a pair of semicircular planar spirals as pancake detector coils, as shown in Figure 13. This detector coils have differential outputs with two symmetrical sensing coils. An IOnic probe produces a kind of spiral path on substrate material, with photolithographic processing.

Based on the configuration, the IOnic probes, when there is no flaw, show symmetric EMFs in both detector coils that differ in polarity and cancel each other out. The EMF planar detector is zero. It is also self-nulling. When there are flaws, the detector becomes unbalanced. Therefore, the EMF appears as a flaw representation that will increase with the increasing length of the flaw. The probe can reduce the influence of the lift-off effect and have high sensitivity, as well as an increase in the S/N ratio, as reported in other research [32,33,34,35].

The probe can be used on a test piece surface that has an angle or is uneven [36]. The operation of the probe is designed to detect flaws like cracks of around 50 μm to 500 μm on the surface of the aluminum test piece of friction stir welding (FSW) joints [37].

#### 5.1.7. Theta Probe (One Pancake Circular Excitation Coil and One Tangential Rectangular Detector Coil)

**Figure 14:**
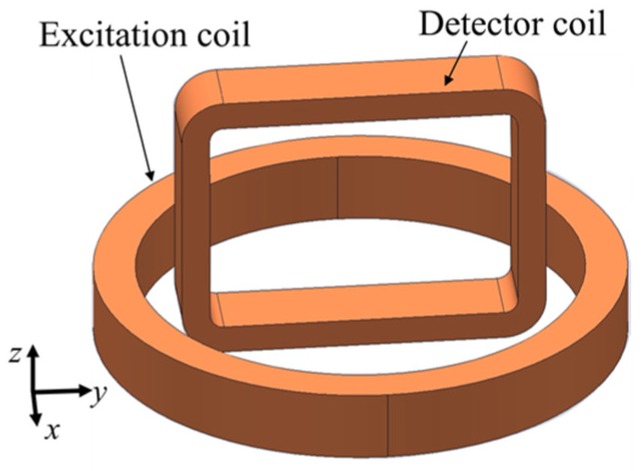
The structure of the Theta probe.

The Theta probe consists of a pancake circular excitation coil and a tangential rectangular detector coil. It has stronger induction and no lift off noise, as reported in other research [38] and as shown in Figure 14. It is different from the general configuration of a UEC excitation probe, in which the excitation coil installed is in a pancake position. However, the detector coil of the probe has similar properties to a tangential rectangular coil which is the same shape as a detector coil of UEC probe. In the configuration, the detector coil only responds to the magnetic field due to flaws like cracks, and it resists lift-off variation, resulting in a high S/N ratio. 

However, when the detector coil is right over the flaw, the detector coil balances with the self-nulling nature [39].

The Theta probe can detect flaws on the test pieces of CFRP, brass and aluminum.

### 5.2. Rotating UEC Probes

#### 5.2.1. Rotating Hoshi Probe (Two Tangential Rectangular Excitation Coils and One Pancake Circular Detector Coil)

**Figure 15:**
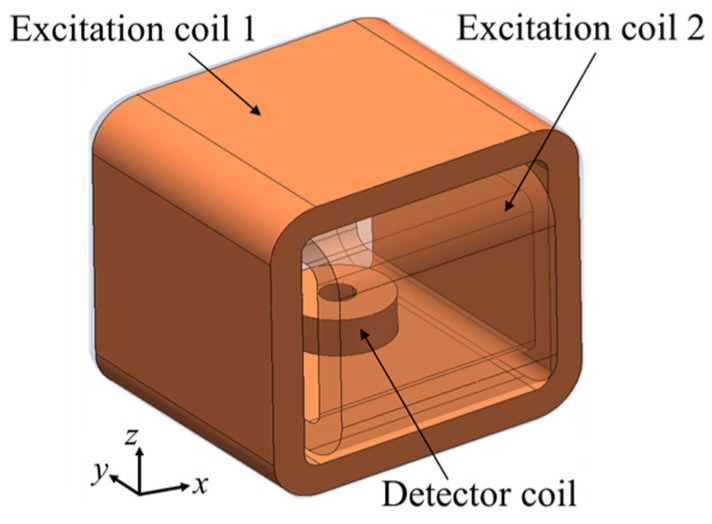
Structure of the Rotating Hoshi probe.

A Rotating Hoshi probe is one of the initial designs of rotating probes. The configuration of a rotating Hoshi probe consists of a pair of tangential rectangular excitation coils arranged orthogonal to each other and shaped like a cube, and a small pancake circular detector coil positioned at the center bottom of the excitation coil [16], as shown in Figure 15.

The rotating UEC is the resultant UEC (*RUEC*) of the UECs generated by excitation coil 1 and excitation coil 2, as shown in the following equation. *UEC*_1_ and *UEC*_2_ respectively are UECs which are generated by excitation coil #1 and excitation coil #2, respectively, by using two excitation currents for which the phase difference is 90°.

**Figure 16:**
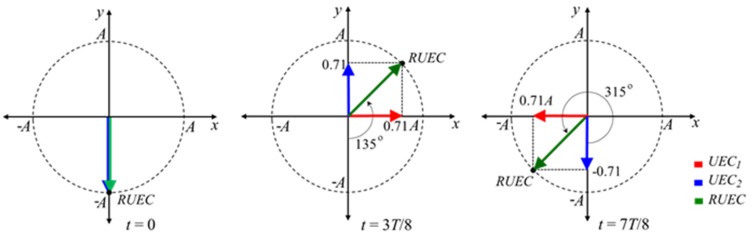
The current flow of the rotating UEC at each phase of the excitation currents.

(2) UEC1=A sin (2πtT)

(3) UEC2=A sin (2πtT)+π2

(4) $$RUEC=\sqrt{{\left(UEC_{1}\right)}^{2}+{\left(UEC_{2}\right)}^{2}}$$

where *T* is the period of the excitation current. *A* is the amplitude of the UECs. The two excitation coils are orthogonally installed. Therefore, it is assumed that *UEC*_1_ generated from excitation coil #1 flows to the *x* direction, and *UEC*_2_ generated from excitation coil #2 flows to the *y* direction, as shown in Figure 16. When two excitation currents are flowing in one period, *RUEC* is rotated in all directions with constant amplitude [40].

The generation principles of the EMF of pancake circular detector coils with flaws and without flaws are the same as that of a one-direction UEC probe. The rotating UEC probe can detect flaws like cracks in all directions on surface of test pieces of steel. In addition, since the rotating UEC allows the flow of eddy currents perpendicular to the length of the flaw, the EMF is maximized. This high sensitivity is one of the main advantages of the probe. However, the scanning time of the rotating UEC is slower than that of the one-direction UEC.

#### 5.2.2. Rotating ACFM Probe (Two Magnetizers, One Combination of a Tangential Rectangular Coil and a Pancake Rectangular Coil of Detector Coil)

**Figure 17:**
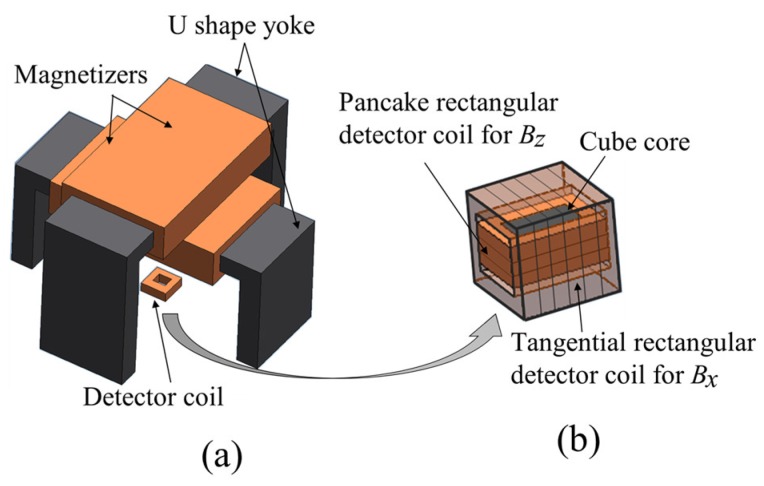
Structure of the rotating ACFM probe: (**a**) rotating ACFM probe, (**b**) detector coil is a combination of tangential rectangular detector coil for $B_{x}$ and pancake rectangular detector coil for $B_{z}$.

The configuration of the probe is two magnetizers which are arranged orthogonally to each other, as shown in Figure 17, and a combination of two detector coils for $B_{x}$ and $B_{z}$, which are the same as the detectors of a one-direction ACFM probe. The two excitation currents for each coil have the same amplitude and a single frequency but are different in phases of 90°. The generation of the rotating UEC with a rotating ACFM probe is same as that of a rotating UEC probe.

The detection principle of flaws with detector coils is the same with that of a one-direction ACFM probe. The advantages and disadvantages are the same as those of the rotating UEC [4,16]. The developed rotating ACFM probe is also suitable for use to detect flaws like cracks on test pieces of steel in an underwater environment [2].

#### 5.2.3. Rotating Dual Driver Planar Probe or IOnic+ Probe (Four Tangential Rectangular Excitation Coils and Four Pancake Quarter Circular Planar Detector Coils)

**Figure 18:**
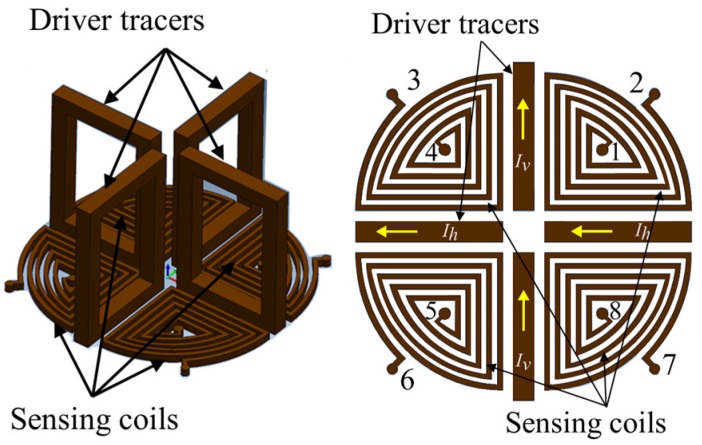
Structure of the IOnic+ probe.

The disadvantage of an IOnic probe is its incapability to detect flaws that are perpendicular to the excitation coil or drive tracer, as reported in other research [37,41]. For this reason, a rotating dual driver planar probe was developed. The configuration is for the drive tracer to be in four parts, arranged like the tangential excitation coils of a rotating UEC, as shown in Figure 18. The four driver tracers (DT) are grouped into two parts, namely the DT horizontal and DT vertical. Each group is connected in series and supplied separately, so that each can set different excitation current amplitudes and phases. The detector coils or sensing coils are also separated into four sections, which occupy four positions of the quadrant—all of which are pancake oriented. The four parts are connected in series into one unit with the connection of terminal pairs 2–3, 4–5, and 6–7. The output from the connection of terminal pairs 1–8 is the differential, which is the total output of the detector coils. This new probe is also known as the IOnic+ probe.

The probe can detect flaws like cracks in all directions on test piece of aluminum. In addition, the proper flow of the UEC against a flaw is generated by the setting of the excitation current amplitude and phase for each excitation coil, enhancing the sensitivity of flaw detection [32,42].

## 6. Summary

The improvement of UEC probes will be required for advanced quantitative measurement. From a brief description of the characteristics of some UEC probes, important factors are indicated that take into consideration the need for increased sensitivity in UEC probe design.

The first factor is the configuration of the excitation coil and detection coil. UEC probe configurations must be developed so that the excitation coil and detection coil generate a larger induction current density and cause an increase in the opposite magnetic field in response to flaws. Another candidate for probe configuration is to create higher S/N ratios in which the signals are generated only when there are flaws and have a high degree of resistance to lift-off variation. 

The second factor is the shape and orientation of the detector coil. A larger interaction zone of the detector coil can measure more opposing magnetic fields, and this increases the EMF. In addition, the self-differential and self-nulling natures of the detector coil should be considered. That is, the shape and orientation of the detector coils should be determined by considering the flows of the opposite magnetic fields from the eddy currents, whether in the shape of a single coil or if it is built from two or several coils. Moreover, advanced magnetic sensors, such as a GMR sensor, a magnetic impedance (MI) sensor [26,43], and a superconducting quantum interference device (SQUID) sensor are also applicable as a detector instead of a detector coil [44,45].

The third factor is improvement of flaw detectability and inspection efficiency. The candidate for this is similar to the rotating UEC probes. To achieve that, not only the configuration of the probe but also the development of the probe control devices is important. The need for control devices is to supply the required variation in excitation currents, such as different amplitudes and phases, so that the probe achieves the maximum sensitivity to flaws in all directions without the mechanical movement of the probe.

Table 1 and Table 2 are summarize the comparison of the models of UEC probes that have been presented.

Finally, the properties of UEC probes were discussed, along with the shapes and orientations of the excitation and detector coils. The advantages and disadvantages of UEC probes were indicated based on their configurations. This review is expected to contribute information to researchers whose work focuses on developing UECT probes. 

## Figures and Tables

**Table 1:** Summary of the comparison of the models of one-direction UEC probes presented.

No	Models and Configurations	Characteristic
1	UEC probe (Hoshi probe) **Excitation;** One tangential rectangular coil.) **Detector;** One pancake circular coil	The probe has self-differential and self-nulling properties and is immune to the lift-off. The probe can detect the flaws on the weld zone surface and the edge of the test piece and can provide phase signal as information of flaw depth. The probe has a relatively weak induction and a small detection signal. The probe cannot detect the direction of flaw length that is parallel to the direction of UEC flow.
2	Cross probe **Excitation;** One tangential rectangular coil **Detector;** One tangential rectangular coil	The probe has self-differential and self-nulling properties and is immune to the lift-off. The probe can detect the flaws on the weld zone surface and can provide phase signal as information of flaw depth. The probe has a relatively weak induction and a moderate detection signal. The probe cannot detect the direction of flaw length that is parallel to the direction of UEC flow.
3	The plus- probe **Excitation;** One tangential rectangular coil **Detector;** Two tangential rectangular coils	The probe has self-differential and self-nulling properties and is immune to the lift-off. The probe can detect flaws on the weld zone surface and can provide phase signal as information of flaw depth. The probe has a relatively weak induction, and a moderate detection signal. The probe cannot detect the direction of flaw length that is parallel to the direction of UEC flow. The probe cannot detect a flaw that is smaller than the distance between the two detector coils on the position right under the excitation coil.
4	UEC probe with a GMR detector **Excitation;** One tangential rectangular coil **Detector;** One GMR component	The probe can detect the flaws on the weld zone surface and provides information of flaw depth. GMR is high accurate. The probe can work at low frequencies below 1 kHz so that it can detect flaws deeper from the surface of the test piece. The probe does not have self-differential and self-nulling properties and is not immune to the lift-off.
5	U-shape ACFM probe **Excitation;** One magnetizer **Detector;** One Combination of a tangential rectangular coil and a pancake rectangular coil	The probe relatively has strong induction and is immune to the lift-off. The probe can detect the flaws through coating and the flaws on the weld zone surface. The probe can provide information on the size and depth of flaw only from amplitude signal of $B_{x}$ and $B_{z}$. The probe cannot detect the direction of flaw length that is parallel to the direction of UEC flow.
6	IOnic probe **Excitation;** One tangential rectangular coil **Detector;** Two pancake semicircular planar coils	The probe has self-differential and self-nulling properties and is immune to the lift-off. The probe has high sensitivity and can detect the micro size flaws on the friction stir welding (FSW) zone surface. The probe can provide information of flaw depth. Probe production requires high precision in making symmetrical planar spiral detectors that must be precisely similar between the two sides.
7	Theta probe **Excitation;** One pancake circular coil **Detector;** One tangential rectangular coil	The probe relatively has strong induction and is immune to the lift-off. The probe can detect the flaws on the weld zone surface and can provide phase signal as information of flaw depth. The probe cannot detect flaws that is shorter than the diameter of the excitation coil.

**Table 2:** Summary of the comparison of the models of rotating UEC probes presented.

No	Models and Configurations	Characteristic
1.	Rotating UEC Hoshi probe **Excitation;** Two tangential rectangular coils **Detector;** One pancake circular coil	The probe has self-differential and self-nulling properties and is immune to the lift-off. The probe can detect flaws in all direction on the weld zone surface and edge of the test piece. The probe can provide phase signal as information of flaw depth. The probe relatively has a weak induction and a small detection signal.
2.	Rotating ACFM probe **Excitation;** Two magnetizers **Detector;** One combination of a tangential rectangular coil pancake rectangular coil	The probe relatively provides strong induction and is immune to the lift-off. The probe can detect flaws through coating, and detect flaws in all direction on the welding zone surface The probe can provide information on the size and depth of flaw only from amplitude signal of $B_{x}$ and $B_{z}$. The probe is not recommended for small test piece.
3.	Rotating dual driver planar probe or IOnic+ probe **Excitation;** Four tangential rectangular coils **Detector;** Four pancake quarter circular planar coils	The probe has self-differential and self-nulling properties and is immune to the lift-off. The probe has high sensitivity and can detect the micro size flaws in all direction on the FSW zone surface. The probe can provide information of flaw depth. The control of driver tracer currents and phase can maximize the detectability of flaws without the change of the probe position. Probe production requires high precision in making symmetrical planar spiral detectors that must be precisely similar between four sides.
